# Village doctors' dilemma in China: A systematic evaluation of job burnout and turnover intention

**DOI:** 10.3389/fpubh.2022.970780

**Published:** 2022-11-10

**Authors:** Yuquan Chen, Yanwei You, Yaying Shen, Zifei Du, Tao Dai

**Affiliations:** ^1^Institute of Medical Information/Medical Library, Chinese Academy of Medical Sciences, Beijing, China; ^2^Peking Union Medical College, Beijing, China; ^3^School of Social Sciences, Tsinghua University, Beijing, China; ^4^Division of Sports Science and Physical Education, Tsinghua University, Beijing, China; ^5^The Affiliated TCM Hospital of Guangzhou Medical University, Guangzhou, China

**Keywords:** turnover intention, job burnout, village doctors, China, systematic review, meta-analysis

## Abstract

**Background:**

Village doctors (VDs) in China undertook arduous primary healthcare missions. However, they received little attention in comparison to doctors in urban public secondary and tertiary hospitals. There is an urgent need to explore the overall situation of turnover intention and job burnout among VDs to evaluate and adjust current health manpower policy.

**Methods:**

In this study, seven databases like PubMed, EMBASE, Web of Science (WOS), WanFang, China Science and Technology Journal Database (VIP), Chinese BioMedical Literature Database (CBM), and China National Knowledge Infrastructure (CNKI) were systematically searched, relevant experts were consulted, and empirical research on job burnout and turnover intention among VDs in international publications was evaluated. Therefore, we evaluated the prevalence of job burnout among VDs in general, across all dimensions and different severity levels, as well as the scores of each category. For turnover intention, we assessed the prevalence of different groups and their overall situation and also identified significant contributors.

**Results:**

In this study, we integrated 20 research evidences on job burnout and turnover intention among 23,284 VDs from almost all provinces in China, and the prevalence of turnover intention among VDs in China was as high as 44.1% [95% confidence interval (CI): 34.1–54.2], which was two to four times that of primary health workers in high-income countries, but not much different from some developing countries. Simultaneously, VDs with the highest risk of turnover intention were men [odds ratio (OR): 1.22 (1.05–1.43)], those with a monthly income below USD 163.4 [OR: 0.88 (0.78–0.98)], those with a high educational level [OR: 0.88 (0.78–0.98)], and those <40 years old [OR: 1.27 (1.16–1.40)]. Similarly, the detection rate of job burnout toward them was 59.8% (95% CI: 38.7–79.1) with the MBI-GS score being 44.44 (95% CI: 37.02–51.86) in a total of 90, while the detection rate of job burnout in moderate and above almost reached 20%. The most significant contributor that affects job burnout was low personal accomplishment (LPA), and the detection rate for moderate and higher severity was 65.2% (95% CI: 58.7–71.7).

**Conclusion:**

Attention should be paid to the high turnover intention and severe job burnout of primary health workers in rural areas of developing countries, and targeted measures should be taken to improve the situation. Health policymakers should increase financial subsidies for VDs, set a reasonable workload, improve various health policies such as pension insurance for VDs, and encourage “targeted training” for medical students to enrich and expand their team.

**Systematic review registration:**

https://www.crd.york.ac.uk/PROSPERO/, identifier: CRD42021289139.

## Introduction

Village doctors (VDs), who are affectionately known as “gatekeepers” of rural health service systems, refer to personnel who have obtained the qualification certificate of VDs and work in village clinics, and are also the main guardians of farmers' health and provide basic public health services, including mainly the establishment of rural health archives, health education, prevention and control of infectious diseases, healthcare for the elderly, the management of chronic diseases, etc. (1, 2). In the mid-1950s, VDs were called as “barefoot doctors,” because they did not have good experience in the professional medical system and were mostly recruited from ordinary villages, and their main workplace was the village clinic funded and operated by the government. However, they were not included in government employees and only in temporary workers who faced dismissal at any time and were at the bottom of China's rural health system (3–5). Nevertheless, the World Health Organization (WHO) still regarded the barefoot doctor system as a successful example of healthcare provision in developing countries that addressed medical resource shortages *via* political mobilization by the government (3). In 1985, the Ministry of Health stopped using the term barefoot doctors; those who passed a government assessment qualified as “VDs” (3, 6). Since then, VDs have undertaken more and more basic healthcare missions gradually and have drawn increasing attention from the academic community, particularly in relation to their training and career development. Researchers have recently started to focus on problems like relatively low pay, job burnout, disparity in human resources, social security, turnover intention, and the limited service capacity of VDs (1, 7). According to the statistical results of the China National Health Commission (8), in 2020, the number of diagnoses and treatments in village clinics reached 1.43 billion, accounting for 18.48% of the total medical service in China. On average, the annual number of diagnoses and treatments in each village clinic was 2,349. VDs play an irreplaceable role in ensuring and improving the health of rural residents as the most basic and extensive medical service providers in rural areas (2).

However, since 1980s, with the collapse of the rural collective economic system in China, former VDs needed to pay for the operation of village clinics. In this case, the privatization of user-paid medical and healthcare system and services led directly to a sharp decline in funding for rural medical and healthcare (9). Consequently, due to the lack of official funding, the medical technology level and service quality of village clinics have lagged behind in recent years. More seriously, the health system reform schedule also excluded VDs from the government project all the time before the New Rural Cooperative Medical System (NRCMS), which was initiated by Chinese Government in 2003, resulted not only in the low satisfaction of rural residents with medical services but also in the high turnover rate of VDs themselves (10). Simultaneously, the government also announced “Deepening the Reform of the Medical and Healthcare System” in 2009. Nevertheless, the shortage of personnel has hindered reforms in the rural healthcare system (6) and this situation has so far increasingly deteriorated. The number of VDs in China significantly decreased from 1.61 million in 2011 to 741,000 in 2020, while their daily average number of diagnoses and treatments increased from 6.7 to 7.6 during the same period [raw data source: the official website of the China National Health Commission (http://www.nhc.gov.cn/)], indicating that the burden of VDs was not reduced but that a significant loss of personnel occurred.

Job burnout refers to a series of psychological and physiological reactions caused by the pressure of the interpersonal relationship and work itself. It is characterized by three dimensions: emotional exhaustion (EE), depersonalization (DE), and low personal accomplishment (LPA) (6), and is affected by work, individual, and organizational and social factors (11, 12). Turnover intention refers to the thought that an individual has to resign from his current job and look for another job (13). In the classical turnover theory, turnover intention is usually regarded as an important cognitive process before turnover behavior. It is the most effective antecedent variable to predict turnover behavior (14, 15). The higher the turnover intention, the greater the probability that an individual will engage in turnover behavior.

In recent years, the increasing trend in job burnout among VDs in China can also be reflected in a lack of enthusiasm and willingness to provide high-level services (16). The primary cause of this phenomenon has been blamed on healthcare system reforms, integrated management, low income, heavy workload, and other determinants, which indirectly hinder health promotion and increase the tendency of VDs to leave (16–18). However, as this group is at the bottom of the rural health system, limited attention has been paid to them. Currently, there are only a few studies on job burnout and turnover intention among VDs in specific areas or in a small range. In contrast, in China, doctors in large hospitals, such as urban secondary or tertiary public ones, not only have much higher social status and welfare benefits than VDs (19–21) but also have drawn much attention in academia, with respect to the evaluation of the current situation or influencing factors of turnover or job burnout toward them (14, 19, 22–24). VDs are also important and need the same attention, but with regard to the studies of two most important factors affecting turnover behavior and work efficiency, namely job burnout and turnover intention, the corresponding original survey was insufficient compared with their colleagues in urban public hospital. Moreover, no scholar has comprehensively evaluated the current dilemma of VDs in China from the perspective of a systematic review. Consequently, our team has undertaken this mission and attempted to close this gap through a systematic evaluation of the current status and potential significant contributors of turnover intention and burnout among VDs in China.

Collectively, this paper aimed to answer the following two key questions:

- What is the current situation of job burnout and turnover intention among VDs in China?
- What are the significant contributors that affect job burnout and turnover intention in this special group?

## Methodology

A meta-based analysis was applied in this study. Compared with traditional literature review or emerging bibliometric analysis (considering that these types of analyses mostly focused on the knowledge map), both systematic review and meta-analysis had a relatively broad horizon of current hotspots and could quantitatively reflect the research status in the field (25–27). This systematic review was conducted in accordance with the Preferred Reporting Items for Systematic Reviews and Meta-Analysis Protocols guidelines (28) and registered with the International Prospective Register of Systematic Reviews (PROSPERO, registration number: CRD42021289139).

### Search strategy

We searched cross-sectional studies on job satisfaction, turnover intention, and job burnout among Chinese VDs that had been published in electronic databases like China National Knowledge Infrastructure (CNKI), WanFang, China Science and Technology Journal Database (VIP), Chinese BioMedical Literature Database (CBM), PubMed, Embase, and Web of Science (WOS). At the same time, experts in the field of social medicine were consulted to achieve supplements and obtain the relevant literature. After pre-screening and consulting expert advice in the field of social medicine and epidemiology, the amount of literature published before 2011 was not only very small, but also the quality of literature could not be guaranteed, so it was not representative. Consequently, the retrieval time limit is set from 01 January 2011 to 01 January 2022. A search strategy was based on a combination of: “rural doctor,” “rural physician,” “VD,” “village physician,” “turnover intention,” “burnout,” etc. Specific literature retrieval strategies of each database can be found in Supplementary Appendix A.

### Study eligibility

Eligible studies were publications that reported the prevalence or questionnaire score and determinants related to turnover intention or burnout among VDs in China. Eligibility criteria included the following: (1) types of studies: original cross-sectional studies (those presenting non-original data, such as reviews, editorials, opinion papers, or letters to the editor, were excluded); (2) types of participants: Chinese VDs; (3) the outcome of burnout measures: Professional Maslach Burnout Inventory (MBI) series job burnout measurement table should be used as a measurement tool. MBI is the most widely used job burnout measurement tool in the world. In empirical research publications related to job burnout, more than 90% of papers and research reports use the MBI scale as a measurement tool (29–31). Simultaneously, the prevalence or score of burnout and the status of all dimensions were reported in this study. According to the provisions of the MBI series questionnaire, the status should include the low, medium, and high level or the dimensions included EE, DE, and low personal compliance. (4) The outcome of the turnover intention measures: the prevalence of turnover intention and related factors should be reported. (5) The studies whose necessary data information was incomplete or missing, or the repeated published ones, should be excluded.

### Data extraction

Firstly, the title information of the relevant literature was retrieved through a retrieval strategy, and Endnote *X9* software was used for literature management. After the duplication process, two reviewers read the title and abstract for preliminary screening according to the inclusion and exclusion criteria, and then further examined the full text to judge the qualification. Disagreements about inclusion criterion were resolved by a third reviewer. For the final selection of qualified literature, two parallel groups independently extracted the research data and made records, including the first author, survey time, survey area, sampling method, turnover intention, burnout, etc.

### Quality assessment

Two reviewers (YC and YS) independently evaluated the risk of bias (ROB) included in this study and cross-checked the results. When the two reviewers had different opinions, the third reviewer (YY) could decide through discussion. The quality of the cross-sectional studies was evaluated by using the 11 items of the observational study quality evaluation standard recommended by US healthcare quality and research institutions (32). The total score was 11 points, and all included studies were grouped based on their scores, which were categorized as good (8–11), moderate (4–7), and poor (0–3). The ROB of the original study was made by reference to quality results.

### Data synthesis and statistical analysis

The primary outcome of this review was the prevalence or score of turnover intention and job burnout among different groups. Prevalence was estimated as the total number of positive cases (i.e., the turnover intention or burnout cases) divided by the total number of participants. For the evaluation of turnover intention, overall consolidation was defined as the evaluation of prevalence, and there was no report of overall turnover intention in the form of a score (33). Simultaneously, significant contributors to the association between factors and turnover intention among VDs were measured as an odds ratio (OR), which was the secondary outcome of this study. Each factor would be analyzed using a meta-combination, the required related variables that were reported in the questionnaire to be the same in diverse included researches, which meant that it was feasible to merge the factor into two groups. In addition, at least three studies related to each factor had to be included in the meta-analysis. However, for job burnout, the prevalence and score would be reported at the same time. Because MBI series questionnaires have been uniformly adopted, the scoring standards are relatively consistent, so the scores reported can be directly combined. Meanwhile, we would conduct a subgroup analysis based on three dimensional characteristics of job burnout, namely, DE, EE, and LPA, and evaluated the main determinants to explore which one would contribute the most to the outcome.

The *meta*-package in *R* software (version 4.0.3, Auckland University, USA) was mainly used for data analysis, and the main outcome was assessed *via* a single-arm analysis. For prevalence or proportion, firstly, the normality test was conducted. If the data did not conform to the normality, they would be transformed by logarithm, logit, or double antisinusoidal transformation. For the evaluation of scores of job burnout or the prevalence of turnover intention, we used the inverse variance weighting method for pooling, and the significance of the pooled OR and its 95% confidence interval (CI) were determined using the *Z*-test when a meta-analysis was conducted.

The Cochrane *Q*-test and *I*^2^ value were used to test whether there was heterogeneity among all studies (34). According to the Meta-analysis of Observational Studies in the Epidemiology guideline (35), if *p* > 0.10 and *I*^2^ ≤ 50%, it was indicated that there was no statistical heterogeneity among the research results, and the fixed effects model was applied to analyze the results; if *p* ≤ 0.1 and *I*^2^ > 50%, the random-effects model was used for meta-analysis. Publication bias was evaluated using Egger's test. Sensitivity analysis was performed by grouping or excluding low-quality studies if necessary. If quantitative synthesis and a meta-analysis were not feasible, a narrative approach and descriptive statistics were used.

## Results

### Study and sample characteristics

**Figure 1:**
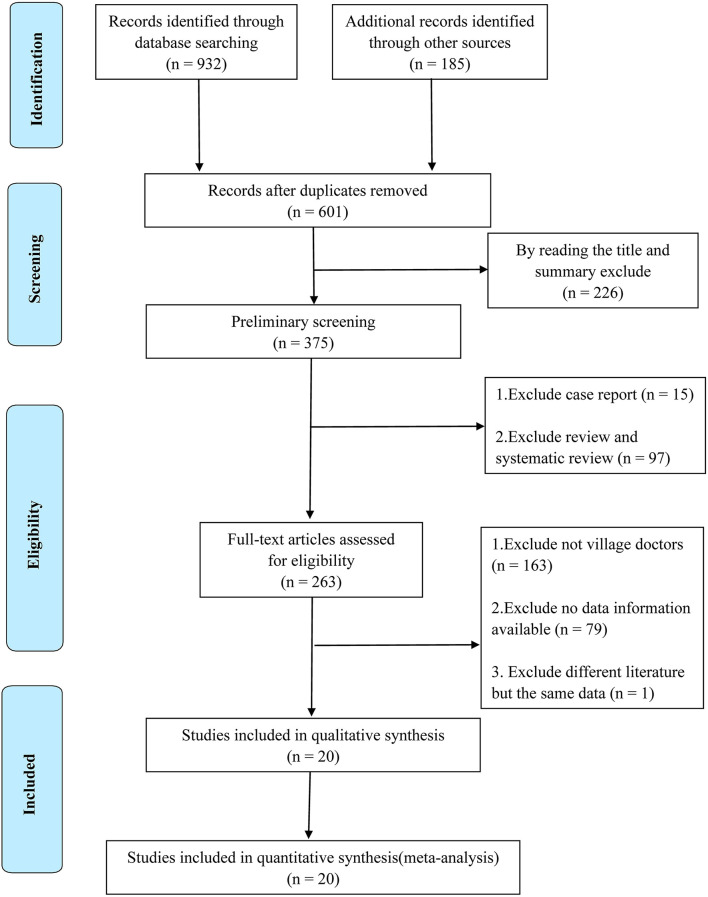
PRISMA flow chart of included studies.

A total of 1,117 literature studies were obtained from various databases and references recommended by experts. Using Endnote *X9* software, 516 duplicate literature studies were eliminated, and by reading titles and abstracts 226 irrelevant literature studies were eliminated. Subsequently, the type of review studies, documents with inconsistent research objects, and incomplete data information were excluded by reading the full text. Finally, a total of 20 literature studies were included for qualitative and quantitative analyses (see Figure 1 for a detailed process).

In the analysis of turnover intention, we included 13 original studies (2, 36–46), including 17,346 VDs. A characteristic of 13 included studies of turnover intention among Chinese VDs is shown in Tables 1, 2. Of these, seven studies were conducted in eastern China (2, 37, 38, 42, 44–46), six in central provinces (36, 38–40, 43, 47) and one in the western region (38). And, these studies were conducted in all 21 provinces of China between 2012 and 2020. A total of seven studies (36–39, 41, 43, 47) used a dichotomous question to measure turnover intention (Do you want to leave your job? Yes/No), six studies used scales [Note: all used the five-point Likert scale, which was collapsed into a binary category of disagree (highly disagree, disagree, and average) and agree (agree and highly agree) to evaluate the status of turnover intention, and people who chose /agree/ were regarded as participants with turnover intention], and all studies reported prevalence. Meanwhile, 11 original studies, including 11,378 VDs, were included in the analysis of job burnout.

**Table 1:** A characteristic of 20 included studies of burnout and turnover intention among Chinese village doctors (VDs).

**Study ID**	**First author**	**Publication year**	**Survey area**	**Investigation period**	**Sampling method**
1	Xu Zhou (48)	2021	Shandong Province	2020.05	Multi stage random sampling
2	Siyu Chen (49)	2019	Shandong Province	2018.01~2018.03	Convenience sampling
3	Ye Wu (50)	2019	Jilin Province	2017.11~2018.01	Multi stage stratified cluster sampling
4	Yun Sun (47)	2017	Anhui Province	2015.09~2017.03	Convenience sampling
5	Li Du (53)	2015	Guizhou Province	*NA*	*NA*
6	Bingjie Shen (36)	2018	Central China	2016.11~2017.04	Census
7	Xinyi Zhao (2)	2021	Various regions of China	*NA*	Convenience sampling
8	Xuewen Zhang (17)	2021	Shandong Province	2019.05~2019.06	Stratified cluster random sampling
9	Xiaodong Yao (51)	2021	Shanxi Province	2019.07~2019.09	Multi stage cluster sampling
10	Jialin Wang (37)	2021	Shandong Province	2020.05	Multi stage random sampling
11	Zhiyuan Li (38)	2021	6 provinces in China	2013~2017	Stratified cluster sampling
12	Hao Li (39)	2020	Shanxi Province	*NA*	Multi stage stratified random sampling
13	Haiming Xie (40)	2015	Hebei Province	2013.11	Stratified cluster sampling
14	Xiaojuan Zhang (41)	2013	A poor county in a mountainous area	*NA*	*NA*
15	Chao Gong (42)	2020	Tianjin	2019	Stratified random sampling
16	Pengqian Fang (43)	2014	Hubei Province	2012.07~2012.08	Multi stage stratified cluster sampling
17	Yue Lu (44)	2018	Shandong Province	2016.10~2016.11	Stratified cluster random sampling
18	Qianqian Yu (45)	2018	Shandong Province	2015.10~2015.11	Multi stage stratified random sampling
19	Haipeng Wang (46)	2020	Shandong Province	2017.12	Multi stage random cluster sampling
20	Yiqing Mao (52)	2020	Hubei and Henan Provinces	2016.12~2017.03	Convenience sampling

NA, not reported.

**Table 2:** A characteristic of 13 included studies of turnover intention among Chinese VDs.

**Study ID**	**Study quality score**	**Sample size (qualified rate %)**	**TI assessment tool**	**Prevalence of turnover intention *N* (%)**
10	10	2,272 (84.4)	Dichotomous question	1,076 (47.36)
11	6	2,554 (82.6)	Dichotomous question	1,541 (60.34)
12	8	254 (92.2)	Dichotomous question	171 (67.32)
6	10	1,669 (100)	Dichotomous question	568 (34.03)
13	10	162 (100)	Michael & Spector Turnover Intention Scale	140 (86.42) (score of >3 out of 5)
14	4	68 (100)	Dichotomous question	10 (14.7)
4	9	379 (95.95)	Dichotomous question	272 (71.77)
15	8	2,652 (93.5)	The self-made 11-item 5-point Likert Turnover Intention Scale	464 (17.5) (score of >33 out of 50)
8	9	2,693 (96.6)	Chinese Turnover Intention Scale	1,263 (46.3)
16	9	1,889 (97.88)	Dichotomous question	695 (36.8)
17	5	1,037 (98.57)	The self-made 10-item 5-point Likert Turnover Intention Scale	498 (48.02) (score of >32 out of 50)
18	6	1,018 (92.5)	Cammann Turnover Intention Scale	265 (26.03) (quite agree and very agree)
19	8	699	The self-made 5-point Likert Turnover Intention Scale	115 (score of >3 out of 5)

A characteristic of the 11 included studies of job burnout among Chinese VDs is shown in Tables 1, 3. These studies were conducted in 19 provinces of China between 2015 and 2020. In the literature studies that defined the scope of this study, five studies were conducted in eastern China (2, 46, 48–50), four in central provinces (36, 47, 51, 52), and one in the western region (53). All studies were based on the MBI series scale.

Table 3A characteristic of 11 included studies of job burnout among Chinese VDs.

**Table:** 

**Study ID**	**Assessment tool**	**Sample size (qualified rate%)**	**Total number of burnout**	**Burnout score (Mean ±SD)**	**NLB**	**NMB**
1	MBI-HSS	2,272 (81.0)	*NA*	*NA*	*NA*	*NA*
2	MBI-GS	316 (98.8)	239	*NA*	145	84
3	MBI-GS	499 (97.84)	325	*NA*	265	57
4	MBI-GS	379 (95.95)	260	38.56 ± 12.56	189	67
5	MBI-GS	759 (81.4)	*NA*	*NA*	*NA*	*NA*
6	MBI-HSS	1,669 (100.0)	*NA*	*NA*	*NA*	*NA*
7	MBI-HSS	1,248 (97.5)	295	*NA*	*NA*	*NA*
8	MBI-GS	2,684 (96.2)	1,762	42.46 ± 21.099	*NA*	*NA*
9	MBI-GS	528 (91.7)	*NA*	52.3 ± 12.7	*NA*	*NA*
19	MBI-HSS	699	*NA*	*NA*	*NA*	*NA*
20	MBI-HSS	325	*NA*	*NA*	*NA*	*NA*

**Table:** 

**NHB**	**LEE**	**MEE**	**HEE**	**SEE**	**LDE**	**MDE**	**HDE**	**SDE**	**LPA**	**MPA**	**HPA**	**SPA**
*NA*	1,021	1,251^*^		19 ± 12.2	1,500	772^*^		*NA*	197	2,075^*^		17.2 ± 13.3
10	87	119	110	*NA*	108	87	121	*NA*	111	93	112	*NA*
3	*NA*	*NA*	*NA*	18.98 ± 8.24	*NA*	*NA*	*NA*	11.62 ± 5.34	*NA*	*NA*	*NA*	14.34 ± 6.66
4	228	126	25	14.13 ± 7.54	95	91	193	11.57 ± 6.36	168	91	120	12.86 ± 6.9
*NA*	574	123	62	11.42 ± 8.31	449	218	92	5.87 ± 6.03	673	86	0	18.82 ± 9.17
*NA*	571	511	587	*NA*	423	980	423	*NA*	479	355	835	*NA*
*NA*	*NA*	*NA*	*NA*	29.61 ± 16.19	*NA*	*NA*	*NA*	4.84 ± 6.74	*NA*	*NA*	*NA*	36.03 ± 10.16
*NA*	1,230	652	811	18.97 ± 12.28	1,778	316	599	5.96 ± 6.913	1,034	359	1,300	17.53 ± 13.42
*NA*	*NA*	*NA*	*NA*	16.6 ± 6.1	*NA*	*NA*	*NA*	13.3 ± 5.2	*NA*	*NA*	*NA*	22.4 ± 6.8
*NA*	*NA*	*NA*	186	*NA*	*NA*	*NA*	39	*NA*	*NA*	*NA*	245	*NA*
*NA*	194	46	85	*NA*	249	25	51	*NA*	176	53	96	*NA*

NLB, total number of people with low burnout; NMB, total number of people with medium burnout; NHB, total number of people with high burnout; LEE, total number of people with low emotional exhaustion; MEE, total number of people with medium emotional exhaustion; HEE, total number of people with high emotional exhaustion; SEE, score of emotional exhaustion (mean ± standard deviation (SD)); LDE, total number of people with low depersonalization; MDE, total number of people with medium depersonalization; HDE, total number of people with high depersonalization; SDE, score of depersonalization (mean ± SD); LPA, total number of people with a low degree of low personal achievement; MPA, total number of people with a medium degree of low personal achievement; HPA, total number of people with a high degree of low personal achievement; SPA, score of low personal achievement (mean ± SD).

* The total number of people with medium and high degrees was summarized.

**Figure 2:**
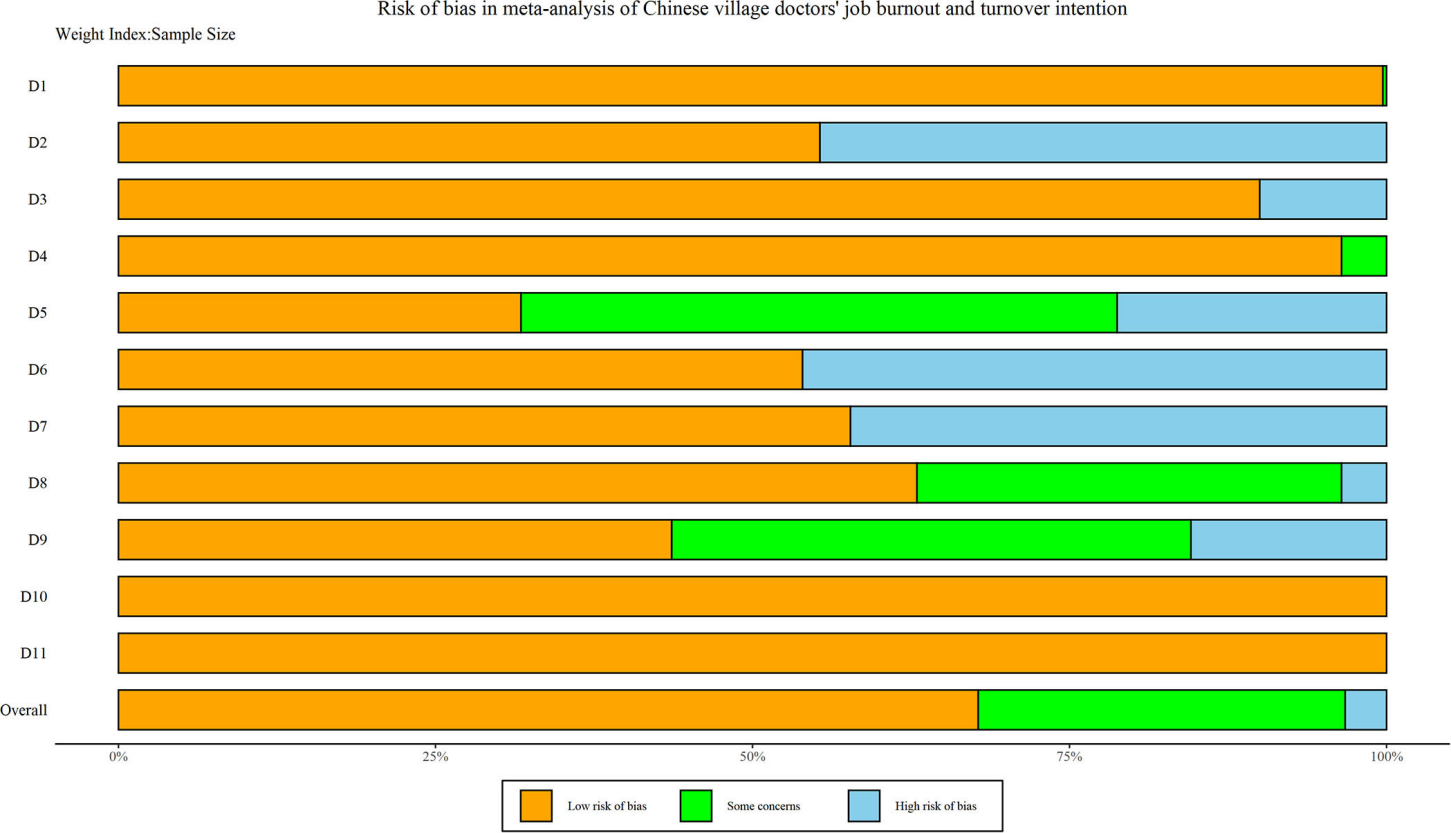
A summary plot of risk bias.

Table 4 demonstrates the quality evaluation of studies from the original literature, including 12 high-quality studies, seven medium-quality studies, and one low-quality study. The average overall study quality score was 7.50 and SD was 2.04. A summary plot of the risk bias assessment of all studies is shown in Figure 2. Simultaneously, the concrete traffic light plot is presented in Supplementary Appendix B. After quality evaluation, it was evident that the overall quality of the original study was relatively high. Therefore, by excluding one low-quality study, other literature studies included in the final study can be directly analyzed qualitatively and quantitatively. For the low-quality study, we intended to use sensitivity analysis to evaluate its effects.

**Table 4:** Quality evaluation results of job burnout and turnover intention among Chinese VDs.

**Study ID**	**First author**	***D1***	***D2***	***D3***	***D4***	***D5***	***D6***	***D7***	***D8***	***D9***	***D10***	***D11***	**Overall**
1	Xu Zhou (48)	1	1	1	1	1	0	1	1	Unclear	1	1	9
2	Siyu Chen (49)	1	0	1	1	Unclear	0	0	1	Unclear	1	1	6
3	Ye Wu (50)	1	0	1	1	0	0	1	1	Unclear	1	1	7
4	Yun Sun (7)	1	1	1	1	Unclear	1	1	1	Unclear	1	1	9
5	Li Du (53)	1	0	0	Unclear	0	0	0	0	Unclear	1	1	3
6	Bingjie Shen (36)	1	1	1	1	1	1	1	Unclear	1	1	1	10
7	Xinyi Zhao (6)	1	0	0	1	1	0	0	1	Unclear	1	1	6
8	Xuewen Zhang (17)	1	1	1	1	Unclear	1	1	Unclear	1	1	1	9
9	Xiaodong Yao (51)	1	1	1	1	Unclear	1	1	Unclear	1	1	1	9
10	Jialin Wang (37)	1	1	1	1	Unclear	1	1	1	1	1	1	10
11	Zhiyuan Li (38)	1	0	1	1	Unclear	0	0	1	0	1	1	6
12	Hao Li (39)	1	0	0	1	1	0	1	1	1	1	1	8
13	Haiming Xie (40)	1	1	1	1	1	1	1	Unclear	1	1	1	10
14	Xiaojuan Zhang (41)	Unclear	0	0	Unclear	1	0	1	0	Unclear	1	1	4
15	Chao Gong (42)	1	0	1	1	0	1	1	1	Unclear	1	1	8
16	Pengqian Fang (43)	1	1	1	1	Unclear	1	0	1	1	1	1	9
17	Yue Lu (44)	1	0	1	1	0	0	0	Unclear	0	1	1	5
18	Qianqian Yu (45)	1	0	1	1	1	0	0	Unclear	Unclear	1	1	6
19	Haipeng Wang (46)	1	1	1	1	1	0	0	Unclear	1	1	1	8
20	Yiqing Mao (52)	1	1	1	1	Unclear	1	0	1	Unclear	1	1	8

D1: define the source of information (survey and record review); D2: list inclusion and exclusion criteria for exposed and unexposed subjects (cases and controls) or refer to previous publications; D3: indicate the time period used for identifying patients; D4: indicate whether or not subjects were consecutive if not population-based; D5: indicate if evaluators of subjective components of the study were masked to other aspects of the status of the participants; D6: describe any assessments undertaken for quality assurance purposes (e.g., test/retest of primary outcome measurements); D7: explain any patient exclusion from analysis; D8: describe how confounding was assessed and/or controlled; D9: if applicable, explain how missing data were handled in the analysis; D10: summarize patient response rates and completeness of data collection; D11: clarify what follow-up, if any, was expected and the percentage of patients for which incomplete data or follow-up was obtained.

### Prevalence and significant contributors of turnover intention among VDs

**Figure 3:**
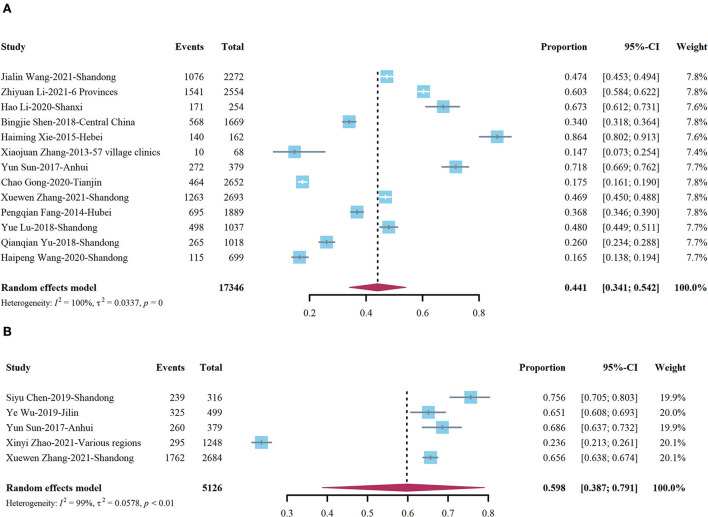
Forest plot of turnover intention **(A)** and job burnout **(B)** among village doctors (VDs).

Table 5 presents the prevalence of turnover intention among VDs in China. The pooled prevalence was 44.1% (95% CI: 34.1–54.2). Egger's test showed no publication bias in the summary results (*p* = 0.2227 > 0.05, *t* = 1.29, intercept = 0.2364). Its forest plot is shown in Figure 3. Xie et al. (40) reported that the highest prevalence was 86.4%, whereas Zhang (41) reported that the lowest prevalence was 14.7%. Subgroup analysis by region showed that the highest prevalence was observed in the central regions (45.6%), followed by eastern China (44.9%); however, there were no significant statistical differences between them (*p* > 0.05). According to the investigation period, a higher prevalence occurred among VDs during 2011–2016 (46.1%), followed by the period 2017–2021 (43.5%), though there were no significant differences between them (*p* > 0.05). With respect to sample size, we selected the midpoint (1,669) of the sample size across all included studies as the critical value, and the prevalence of turnover intention was higher in studies having a sample size <1,669 (47.3%) than in those with a sample size ≥1,669 (40.5%). Simultaneously, subgroup analysis using the survey method showed that the highest prevalence was observed using a non-probabilistic scheme (63.7%), followed by random sampling (38.4%) and census (34.0%), even if the census only contained one study and 1,669 VDs.

**Table 5:** Prevalence of turnover intention among VDs in China.

**Variables**	**Characteristic**	**Included studies**	**Prevalence (95% *CI*)**	***Q*-test (*I^2^*) (%)**	***p*-Value**
Overall		13	0.441 (0.341–0.542)	99.5	–
By region	East	8	0.449 (0.312–0.586)	99.6	0.913
	Central	3	0.456 (0.332–0.581)	98.2	
By sample size	<1,669	7	0.473 (0.282–0.663)	99.4	0.017
	≥1,669	6	0.405 (0.27–0.539)	99.7	
By gender	Male	5	0.469 (0.365–0.573)	98.6	0.045
	Female	5	0.418 (0.319–0.516)	96.9	
By survey method	Random sampling	7	0.384 (0.258–0.51)	99.5	0.009
	Non-probabilistic sampling	4	0.637 (0.452–0.823)	99.4	
	Census	1	0.34 (0.318–0.364)^*^	–	
By period	2011~2016	3	0.461 (0.098–0.824)	99.4	0.687
	2017~2021	10	0.435 (0.321–0.549)	99.6	
By age	<30	5	0.49 (0.41–0.57)	51	0.056
	30~40	5	0.486 (0.37–0.602)	97	
	>40	4	0.454 (0.351–0.557)	98.1	
By income	<163.4 USD	3	0.528 (0.480–0.570)	73.7	0.022
	163.5–490.1 USD	3	0.465 (0.423–0.507)	83.2	
	>490.1 USD	3	0.395 (0.297–0.514)	76.6	
By educational level	Low^**^	4	0.371 (0.349–0.393)	21.9	0.034
	Medium^**^	5	0.475 (0.367–0.584)	98.2	
	High^**^	5	0.527 (0.386–0.667)	96.9	

* Clopper–Pearson confidence interval (CI).

** Low: junior high school and below; Medium: high school and technical secondary school; High: junior college or above [excluding the study of Fang et al. (43)].

In addition, we also clarified the prevalence of turnover intention among VDs in China according to the different demographic characteristics. Firstly, subgroup analysis by gender showed that a higher prevalence was observed among male VDs (46.9%), followed by female VDs (41.8%), combined with a significant difference between two different genders (*p* < 0.05). Secondly, a Classification and summary were done according to the age of VDs at the time of investigation, whose turnover intention was from 45.4 to 49% in the different age groups. Furthermore, according to the monthly income level, the highest prevalence of turnover intention was in VDs whose income level remained below USD 163.4 (52.8%), followed by USD 163.5–490.1 (46.5%) and greater than USD 490.1 (39.5%), and statistically significant differences between them were presented. Finally, we reported the prevalence by different kinds of educational level, and VDs who had a high educational level had the highest turnover intention with 52.7%, followed by the medium level (47.5%) and the low level (37.1%). In particular, excluding one study (43), other studies defined the low education level as junior middle school and below, the medium education level as senior high school and technical secondary school, and the high education level as junior college and above. In one study (36), low educational level was defined as secondary technical school and below with the prevalence of 36.9%, while senior high school was the medium educational level with the prevalence of 36.5%, and the high educational level was defined as bachelor or master and above, whose prevalence was 35%. High heterogeneity was observed across the included studies due to inconsistency of research sites, regions, and objects; indeed, the results of a meta-analysis of the detection rate itself would be very heterogeneous (33, 54, 55). Only one study reported the prevalence of turnover intention among VDs after the COVID-19 pandemic, which was 47.36% (37).

As for significant contributors of turnover intention among VDs, they were examined in six studies (33–36, 40, 41), whose reports meet the metaconsolidation criteria for as mentioned in the data synthesis. Gender [male vs. female, OR: 1.22 (1.05–1.43)], income [below 163.4 USD vs. >163.4 USD, OR: 3.06 (1.94–4.82)], educational level [low or medium educational level vs. high educational level, OR: 0.88 (0.78–0.98)], and age [below 40 years old vs. ≥40 years old, OR: 1.27 (1.16–1.40)], which presented that VDs with the higher risk of turnover intention were men, those with monthly income below 163.4 USD, those with a high educational level and below 40 years of age. Forest plots on each of these contributors can be found in Supplementary Appendix C.

### The prevalence and a significant characteristic contributor of job burnout among VDs

The prevalence and score of job burnout and all its dimensions among them are shown in Table 6. According to our evaluation results, all surveys (2, 6, 36, 46–53) on the job burnout status among VDs were based on the Chinese revised versions of the Maslach Burnout Scale general scale (MBI-GS) (29) and Maslach Burnout Scale—Human Services Survey (MBI-HSS) (56), both of which were widely used in the evaluation of job burnout among Chinese health workers (30, 57–59). The two types of questionnaires included three dimensions: EE, DE, and personal achievement. The EE subscale mainly measured the psychological and physiological extreme fatigue caused by individual emotional and emotional excessive pay, while the DE subscale mainly measured how individuals treated work with a negative and indifferent attitude or emotion. The personal achievement subscale mainly measured individuals' subjective evaluation of the value of work and of themselves (47). Answers to both types of questionnaires were seven Likert score ranging from 0 (never) to 6 (daily). Among them, the higher the score of EE, DE, and low personal achievement (reverse score), and the heavier the degree of burnout (2). In addition, the Cronbach's α of MBI-GS is between 0.79 and 0.94 (2), which had high reliability and validity in China and was further revised and improved by relevant scholars (59–61). The three dimensions of EE, DE, and personal achievement contained five, four, and six entries, respectively, and potential scores range from 0 to 30, 0 to 24, and 0 to 36, respectively. For the MBI-HSS scale (6, 36, 48), the three dimensions of EE, DE, and personal achievement contained nine, five, and eight items, respectively, and potential scores ranged from 0 to 54, 0 to 30, and 0 to 48, respectively.

**Table 6:** Prevalence and score of job burnout and relevant dimensions among VDs in China.

**Variables**	**Characteristic**	**Included studies**	**Prevalence (95% *CI*)**	**Score (95% *CI*)**	***Q*-test (*I^2^*) (%)**
Overall burnout		5	0.598 (0.387–0.791)		99.5
Overall burnout score	MBI-GS	3		44.44 (37.02–51.86)	99.4
By severity	Low	3	0.499 (0.458–0.54)		50.9
	Medium	3	0.184 (0.101–0.267)		93.1
	High	3	0.013 (0.002–0.024)		67.3
Emotional exhaustion	Low	7	0.497 (0.391–0.603)		99
	Medium	6	0.258 (0.198–0.318)		96.2
	High	7	0.239 (0.142–0.336)		99
	Medium + High	6	0.508 (0.456–0.56)		99
Score	MBI-HSS	2		24.3 (13.9–34.7)	99.8
	MBI-GS	5		16.02 (13.15–18.9)	99.2
Depersonalization	Low	7	0.504 (0.347–0.661)		99.6
	Medium	6	0.264 (0.09–0.438)		99.6
	High	7	0.241 (0.154–0.328)		98.9
	Medium + High	6	0.527 (0.475–0.579)		99.7
Score	MBI-HSS	1		4.84 (4.47–5.21)	-
	MBI-GS	5		9.66 (6.41–12.91)	99.7
Low personal achievement	Low	7	0.426 (0.194–0.658)		99.9
	Medium	6	0.19 (0.144–0.236)		95
	High	7	0.329 (0.095–0.563)		99.9
	Medium + High	6	0.652 (0.587–0.717)		99.9
Score	MBI-HSS	2		26.61 (8.16–45.07)	100
	MBI-GS	5		17.19 (14–20.39)	99.3

Among the studies concerning the total detection rate of job burnout, four items were based on the MBI-GS scale (2, 47, 49, 50). The definition of job burnout was at least one of the three dimensions exceeded the critical value. If the scores of the three dimensions were lower than the critical value, it was defined as no burnout. The rest of the study was based on the MBI-HSS scale (6), and its criterion for judging job burnout was “a high EE score (≥27) along with a high DE score (≥13), or a low personal achievement score (≤31).” According to our evaluation results, the overall detection rate of job burnout among VDs in China is 59.8% (95% CI: 38.7–79.1). Egger's test showed no publication bias in the summary results (*p* = 0.8623 > 0.05, *t* = 0.19, intercept = 0.7985). The forest plot is shown in Figure 3. Among the quantifiable results, three studies (2, 47, 51) reported the overall score of job burnout. These studies were designed using the MBI-GS scale, with a total score of 90, and the answers for the items were seven Likert score ranging from 0 (never) to 6 (daily). The higher the score, the stronger the job burnout. According to the meta combination results, the overall score of job burnout was 44.44 (95% CI: 37.0–51.86). Then, we conducted a meta-analysis based on the three studies that reported the severity of job burnout among VDs based on the MBI-GS scale (47, 49, 50). These studies defined mild burnout as a score higher than the critical value in a certain dimension. If the score of some two dimensions was higher than the critical value, it was moderate burnout, while the score for all three dimensions was higher than the critical value, which was high burnout. According to our combined results, the detection rate of low job burnout was 49.9% (95% CI: 45.8–54.0), the detection rate of moderate job burnout was 18.4% (95% CI: 10.1–26.7), and the detection rate of high job burnout was 1.3% (95% CI: 0.2–2.4).

In addition, we also made a detailed evaluation and report on the three dimensions of EE, DE, and personal achievement shared by all studies. Of the six studies (2, 36, 47–49, 53) that reported the number of people detected with different severity of these three dimensions, four were based on the MBI-HSS questionnaire (36, 46, 48, 52). According to the abovementioned criteria, we conducted a meta-analysis of studies that reported detection rates for three dimensions with different severity (2, 36, 47–49, 53). We found that the detection rate of medium and high degrees EE among VDs in China was as high as 50.4% (95% CI: 39.8–61), medium and high degrees of DE was 51% (95% CI: 31.4–70.6), and medium and high degrees of low personal achievement was 57.5% (95% CI: 34.3–80.6). Detailed meta-consolidation rates of the three dimensions of different severity levels are shown in Table 6. After excluding a low-quality study (53), the detection rates of moderate and high degrees of EE, DE, and low personal achievement became 50.8% (95% CI: 45.6–56), 52.7% (95% CI: 47.5–57.9), and 65.2% (95% CI: 58.7–71.7), respectively. Simultaneously, we could clearly find that it was low personal achievement that contributed the most for job burnout and a significant difference was observed among these three groups (*p* < 0.05) through subgroup analysis. Furthermore, we also summarized only studies that reported scores for different dimensions (2, 6, 47, 48, 50, 51, 53). Due to the different criteria for judging the scores of the different scale tools, we conducted a meta-analysis based on the MBI-GS and MBI-HSS scales. The summary results of the scores of the other two dimensions are shown in Table 6.

## Discussion

This study systematically summarized and evaluated the prevalence of turnover intention and job burnout among Chinese VDs in global publications for the first time, and evaluated significant contributors. Our evaluation results showed that the overall prevalence of turnover intention among VDs in China was as high as 44.1%, indicating that almost half of them wanted to leave their current occupation. The high turnover intention among VDs in China was almost two to four times than the high turnover intention among VDs in several high-income countries, which caused a widespread concern. A survey of 1,174 primary care doctors aged 50 years and under in the UK by Hann et al. (62) found that only 11.8% of this group had a high turnover intention. Another survey of 2,263 physicians in the USA showed that only 18.4% of them considered to leave their current job (63). Similarly, in a survey of 23,159 nurses in 385 hospitals in 10 European countries, Heinen et al. (64) found that the proportion of them with a significant turnover intention was only 9%, and the figure was between 5 and 17% in different countries. A study in Japan showed that even after being harassed by patients, doctors' turnover intention was only 17.1% (65). However, the prevalence of turnover intention among doctors in some countries was also relatively high, but generally speaking, it was still lower than that of VDs in China. For instance, a survey of 2,719 doctors in Korea from 2016 to 2017 showed that 30.5% (66) had a high turnover intention. However, compared to various developing countries, this proportion seemed to be little different. For example, a survey of rural nurses in the Philippines found that almost half of them considered to leave their jobs (67), while another report from South Africa showed that this proportion was also as high as 51.1% (68).

Our study also found that approximately six in 10 VDs experienced job burnout, with up to 20% of this group having medium or higher severity. Compared to health workers in other countries, this proportion was also prominent. For example, in a survey of urologists in England, the incidence rate of job burnout was only 28.9% (69). A survey of Polish medical personnel conducted in 2018–2019 showed that the average MBI scale score was 36.08, which was much lower than our results (70). According to the resource conservation theory, the individual's own resources are relatively limited. When there is a potential threat from an external environment or the resources are not supplemented accordingly, individuals experience pressure. In the long run, it is easy to produce job burnout. Resignation is the most common behavior of individuals to deal with job burnout and protect their physical and mental resources. Many studies in China and in other countries showed that job burnout among medical workers was closely related to turnover intention, and there was a significant positive correlation between them, that is, the higher the degree of job burnout, the stronger the turnover intention (46, 54, 71, 72). A study on the relationship of job burnout and turnover intention among medical workers covering 25 provincial administrative regions from 2007 to 2020 in China showed that the *R*-value of the correlation coefficient between job burnout and turnover intention reached 0.43, indicating a high correlation effect (8). Job burnout not only led to a loss of enthusiasm for work, alienation from the organization and work, and increased the degree of turnover intention, but also lead to the decline of work quality and efficiency, which directly threatened the construction of the originally weak rural primary healthcare system (30, 54, 73). Simultaneously, job burnout was also one of the important causes of serious physiological problems such as hypercholesterolemia, type 2 diabetes, coronary heart disease, hospitalization for cardiovascular disorders, and musculoskeletal pain (73). Our evaluation results on the three dimensions of job burnout showed that although the detection rate of each dimension of medium and high degrees was >50%, the proportion of medium and high degrees of low personal achievement was significantly high (57.5%). This result was consistent with the conclusion of a systematic review of primary care nursing, that is, low personal achievement was also the most important factor affecting job burnout among nurses in primary medical institutions (74). Nevertheless, this proportion was still considerably lower than that of VDs in China (31%). The score of low personal achievement measured by O'Kelly et al. (69) using the MBI-HSS scale was 17.1, which is much lower than 26.61 of VDs. This phenomenon was brought on not only by the low social status of VDs but also by the fact that VDs served as “gatekeepers” for patients, making them more likely to be under higher pressure than doctors in urban hospitals in case of adverse medical events (such as workplace violence), and the impact on their low personal achievement would be more evident (54, 75). Likewise, due to the large proportion of mild patients in many hospitals at grass roots level, the low admission rate greatly increased the working hours and workload of VDs, which made low personal achievement less and worse (36, 37).

This systematic review also demonstrated that education, gender, income, and age were significant contributors that affected turnover intention among VDs. It is well-established that VDs with a higher educational level may have higher a turnover intention because VDs with a higher education level had more career choices and promotion opportunities. Several studies also showed that (36, 37, 39, 46, 47), the overall educational level of VDs was low, even most of them were below undergraduate. However, compared to doctors in urban three-level public hospitals in China, the educational level of this group improved significantly (22, 75). This might mean that, under the same conditions, if VDs had a higher educational level, they would have a tendency to go to higher-level hospitals rather than remaining at the very bottom of the rural medical system. The low educational level might further weaken the medical level of the already scarce medical resources in the village. Income was undoubtedly another important factor affecting turnover intention. We noted that the lower the income, the higher the turnover intention. With the advancement of medical reforms, especially since the implementation of the “zero difference” sales of drugs and the equalization of basic public health services, the workload of VDs increased greatly, but the basic drug subsidies and public health subsidies did not increase significantly (16, 17, 76, 77), resulting in their income level not rising but falling, which greatly improved turnover intention among this group. Similarly, gender was also one of the factors affecting turnover intention. Turnover intention among male VDs was significantly greater turnover intention among women, which meant that female VDs might pursue “job stability (37)” to a certain extent. It was interesting to note that VDs below 40 years of age also tended to choose to leave their current job, which might be explained by the fact that the majority of the younger VDs had more choices to make an occupational career decision. Though a small part of this population had turnover behavior for reasons such as lack of interest or severe workload, most of them might still choose it based on income and social status (78). Similarly, in job burnout, low personal achievement become the most important factor affecting the occurrence and severity of job burnout among VDs, as mentioned above. Compared with the other ordinary occupational groups in China, medical work is a high-risk occupation, which has the characteristics of urgent working hours, high task intensity, high-risk nature of work, and high mental and occupational pressure (79). If VDs choose this career not for intense passion, in a context of high mental and occupational pressure, coupled with low salary and poor welfare, they would lose enthusiasm and patience for work and generate negative emotions about their career, which lead to their low sense of professional identity, low personal achievement, and increased job burnout (2). Consequently, VDs should construct reasonable and achievable career expectations, improve time management skills, and participate in psychological counseling programs to mitigate their anxiety. Simultaneously, the government should guide the masses toward developing a correct understanding of VDs, clarify the indispensable role of VDs, improve their sense of professional respect for VDs, so as to improve residents' trust in village clinics and cooperation with their work and enhance the harmonious service relationship between VDs and patients, which will be beneficial to improving the sense of professional belonging and personal achievement for VDs. It should be noted that, in a context of the COVID-19 pandemic, the prevalence of job burnout and turnover intention among VDs might be higher, but few people conducted relevant surveys during this period. A survey showed that, after the COVID-19 pandemic, 65% of medical staff increased their working hours and worked more than 48 h a week, but their treatment did not improve much and their income even decreased slightly (80), which undoubtedly made the situation worse.

Therefore, according to our research results, high turnover intention and severe job burnout among VDs will inevitably aggravate the turnover rate of this group, which plays an indispensable role in rural residents, and their requirement has not decreased. Hence, we strongly suggest that the government should increase financial subsidies for VDs to ensure that their income level can be equal to the average income level of local village cadres, teachers, and other occupations (81). Simultaneously, the government should improve the working environment for VDs, further strengthen the construction of standardized village clinics, and enhance the participation of VDs in decision-making. Superior departments or institutions should fully consider the opinions and ideas of VDs when making decisions related to VDs to reduce their unnecessary work pressure. By standardizing the management mechanism of village clinics and the performance evaluation policy of township health centers for village clinics and reasonably setting the workload, it is ensured that the subsidies are paid in full and on time by ensuring the work quality of VDs and encouraging their work enthusiasm (82). The government should speed up the improvement of the old-age insurance policy for VDs, and guarantee that the old-age insurance level of rural doctors is higher than that of ordinary farmers, so as to reflect their contribution to the development of health undertakings and the important missions they are currently undertaking in the rural health service system (77, 81, 82). Concurrently, the health administrative department should formulate preferential policies to encourage and absorb medical college graduates to serve in the village, implement the “directional” training mode, sign contracts with students interested to work in the village clinic, train the service talents in the village clinic, and ensure the reserve force of VDs, so as to reasonably adjust their age structure and enrich and expand the team of VDs (83).

### Strengths and limitations

This was the first study to systematically evaluate the current status and striking influencing factors of turnover intention and job burnout among Chinese VDs in global publications. Compared to their colleagues in urban public hospitals, extremely less attention was paid to this specific population, who has undertaken a series of vital and Herculean tasks in the rural medical health service system. Our results further indicated that the high rate of significant turnover intention and severe job burnout were exceedingly worrying in comparison to their colleagues in urban public hospitals or primary healthcare workers in other nations or countries, and the potential significant contributors that lead to this dilemma were also recognized. However, some limitations of this study should also be clarified. Firstly, even though there were only two kinds of measurement methods to test turnover intention in included original research in total (a dichotomous question to measure and the five-point Likert scale), due to inconsistencies in research sites, regions, and even the measurement, high heterogeneity was observed across the consolidated results. Hence, future researchers should try to use unified measurement tools. Secondly, a relatively older approach was applied to estimate the prevailing rates of burnout (low, medium, and high) in this review, which has been replaced with a profile approach by Maslach and Leiter (84–86) in many measurements directed at healthcare professionals such as nurses. Yet, due to the nature of research reviewed in this study toward the exploration of job burnout among VDs, all adopt this methodology to ensure the authenticity of the results as much as possible. This method was also compelled to use to reflect the severity of the dilemma of job burnout among VDs, indicating that researchers should use the latest international measurement standards to probe job burnout among VDs in the future. Last but not least, as only Chinese studies are included, the popularization in this study may have limitations. However, although the Chinese population has the bias of sample source, China's VD system has a long history and is also representative to some extent (3).

## Conclusion

Village doctors in China carried out pivotal primary healthcare missions, but in recent years, the situation of staff turnover among this group was very serious. Compared to colleagues in public secondary and tertiary hospitals, little attention was paid to this group. In this study, several core conclusions were summarized as follows: (i) almost half of VDs wanted to leave their current job, which was significantly higher than primary health workers in other high-income countries but not significantly different from some developing countries. Similarly, the severity and proportion of job burnout among VDs were also alarming, with the detection rate in moderate and above reaching almost 20%. (ii) Men with monthly income below USD 163.4, a high educational level, and less than 40 years of age were the important contributors that affected turnover intention among this group. Simultaneously, the most significant contributors that affect job burnout was LPA. Health policymakers should increase financial subsidies for VDs, reasonably set the workload, improve various health policies such as pension insurance for VDs, and encourage “targeted training” of medical students, so as to enrich and expand their team.

## Data availability statement

The original contributions presented in the study are included in the article/Supplementary material, further inquiries can be directed to the corresponding author.

## Author contributions

Conceptualization and writing—original draft preparation: YC. Methodology and data analysis: YC and YY. Material search: YS and ZD. Data extraction: YS, ZD, and YC. Writing—review and editing: YC, YY, YS, ZD, and TD. Supervision, project administration, and funding acquisition: TD. All authors have read and agreed to the published version of the manuscript.

## Funding

Medical and Health Science and Technology Innovation project of Chinese Academy of Medical Sciences: research on the evaluation of Medical Science and Technology Innovation and construction of health service system (2016-I2M-3-018). The funding bodies played no role in the design of the study, data collection, analysis, and interpretation, and in manuscript writing.

## Conflict of interest

The authors declare that the research was conducted in the absence of any commercial or financial relationships that could be construed as a potential conflict of interest.

## Publisher's note

All claims expressed in this article are solely those of the authors and do not necessarily represent those of their affiliated organizations, or those of the publisher, the editors and the reviewers. Any product that may be evaluated in this article, or claim that may be made by its manufacturer, is not guaranteed or endorsed by the publisher.
