# Supplementary material for: Village doctors' dilemma in China: A systematic evaluation of job burnout and turnover intention
Source: Front Public Health. 2022 Nov 10;10:970780. doi: 10.3389/fpubh.2022.970780 (PMC9684668; doi:10.3389/fpubh.2022.970780)
Supplement: Supplementary file 1 [file Data_Sheet_1.DOCX]

**Appendix A. Literature search strategies**

**1. Database:** **China national knowledge infrastructure(CNKI)<2011 January 01 to 2021 December 01>**

[**https://kns.cnki.net/kns8/AdvSearch?dbprefix=SCDB&&crossDbcodes=CJFQ%2CCDMD%2CCIPD%2CCCND%2CCISD%2CSNAD%2CBDZK%2CCCJD%2CCCVD%2CCJFN**](https://kns.cnki.net/kns8/AdvSearch?dbprefix=SCDB&&crossDbcodes=CJFQ%2CCDMD%2CCIPD%2CCCND%2CCISD%2CSNAD%2CBDZK%2CCCJD%2CCCVD%2CCJFN)

Search Strategy:

--------------------------------------------------------------------------------

((SU%='乡村'+'农村'+'基层'+'县乡') AND (SU%='医生'+'医师'+'医务人员'+'赤脚医生') AND (SU%='离职意向'+'离职倾向'+'离职意愿'+'工作意愿'+'留职意愿'+'留职意向')) OR ((SU%='乡村'+'农村'+'基层'+'县乡') AND (SU%='医生'+'医师'+'医务人员'+'赤脚医生') AND (SU%='职业倦怠'+'情绪衰竭'+'去人格化'+'低成就感')) (175)

**2. Database: WANFANG<2011 to 2021>**

[**https://s.wanfangdata.com.cn/advanced-search/paper**](https://s.wanfangdata.com.cn/advanced-search/paper)

Search Strategy:

--------------------------------------------------------------------------------

((主题:"乡村" or "农村" or "基层" or "县乡") and (主题:"医生" or "医师" or "医务人员" or "赤脚医生") and (主题:"离职倾向" or "离职意愿" or "工作意愿" or "离职意向" or "留职意愿" or "留职意向")) or ((主题:"乡村" or "农村" or "基层" or "县乡") and (主题:"医生" or "医师" or "医务人员" or "赤脚医生") and (主题:"职业倦怠" or "情绪衰竭" or "去人格化" or "低成就感")) (166)

**3. Database: China Science and Technology Journal Database (VIP)<2011 to 2021>**

[**http://qikan.cqvip.com/Qikan/Search/Advance?from=index**](http://qikan.cqvip.com/Qikan/Search/Advance?from=index)

Search Strategy:

--------------------------------------------------------------------------------

(M=(乡村 OR 农村 OR 基层 OR 县乡) OR R=(乡村 OR 农村 OR 基层 OR 县乡)) AND (M=(医生 OR 医师 OR 医务人员 OR 赤脚医生) OR R=(医生 OR 医师 OR 医务人员 OR 赤脚医生)) AND (M=(离职倾向 OR 离职意愿 OR 工作意愿 OR 离职意向 OR 留职意愿 OR 留职意向) OR R=(离职倾向 OR 离职意愿 OR 工作意愿 OR 离职意向 OR 留职意愿 OR 留职意向)) OR (M=(乡村 OR 农村 OR 基层 OR 县乡) OR R=(乡村 OR 农村 OR 基层 OR 县乡)) AND (M=(医生 OR 医师 OR 医务人员 OR 赤脚医生) OR R=(医生 OR 医师 OR 医务人员 OR 赤脚医生)) AND (M=(职业倦怠 OR 情绪衰竭 OR 去人格化 OR 低成就感) OR R=(职业倦怠 OR 情绪衰竭 OR 去人格化 OR 低成就感)) (149)

**4. Database:** **Chinese BioMedical Literature Database(CBM)<2011 to 2021>**

<http://www.sinomed.ac.cn/zh/advancedSearch.jsp>

Search Strategy:

--------------------------------------------------------------------------------

((乡村 OR 农村 OR 基层 OR 县乡) AND (医生 OR 医师 OR 医务人员 OR 赤脚医生) AND (离职倾向 OR 离职意愿 OR 工作意愿 OR 离职意向 OR 留职意愿 OR 留职意向)) OR ((乡村 OR 农村 OR 基层 OR 县乡) AND (医生 OR 医师 OR 医务人员 OR 赤脚医生) AND (职业倦怠 OR 情绪衰竭 OR 去人格化 OR 低成就感)) (130)

**5.Database: Pubmed**

<https://pubmed.ncbi.nlm.nih.gov/advanced/>

Search Strategy:

--------------------------------------------------------------------------------

#1 Chinese[Title/Abstract] (245,922)

#2 (China[MeSH Terms]) OR China[Title/Abstract] (333,093)

#3 #1 OR #2 (482,158)

#4 (Health worker[MeSH Terms]) OR Health worker[Title/Abstract] (373,860)

#5 Health officer[Title/Abstract] (527)

#6 (Doctor[MeSH Terms]) OR Doctor[Title/Abstract] (212,813)

#7 (Physician[MeSH Terms]) OR Physician[Title/Abstract] (335,222)

#8 (Medical Personnel[MeSH Terms]) OR Medical Personnel[Title/Abstract] (571,683)

#9 (Medical worker[MeSH Terms]) OR Medical worker[Title/Abstract] (241,717)

#10 (Medical staff[MeSH Terms]) OR Medical staff[Title/Abstract] (39,708)

#11 #4 OR #5 OR #6 OR #7 OR #8 OR #9 OR #10 (848,055)

#12 Rural[Title/Abstract] (156,782)

#13 Countryside[Title/Abstract] (1,575)

#14 District[Title/Abstract] (74,278)

#15 Basic[Title/Abstract] (373,369)

#16 Fundamental[Title/Abstract] (205,116)

#17 Primary[Title/Abstract] (1,692,589)

#18 Grass roots[Title/Abstract] (776)

#19 #12 OR #13 OR #14 OR #15 OR #16 OR #17 OR #18 (2,423,967)

#20 (Turnover Intention[MeSH Terms]) OR Turnover Intention[Title/Abstract] (972)

#21 (Departure Intention[MeSH Terms]) OR Departure Intention[Title/Abstract] (67)

#22 (Demission Intention[MeSH Terms]) OR Demission Intention[Title/Abstract] (2)

#23 (Leave Intention[MeSH Terms]) OR Leave Intention[Title/Abstract] (472)

#24 intent to leave[Title/Abstract] (351)

#25 #20 OR #21 OR #22 OR #23 OR #24 (1500)

#26 (burnout OR “burned out” OR depersonalization or "emotional exhaustion" OR burnout, professional [MESH] OR emotional stress [MESH] OR psychological stress [MESH] OR stress,psychological [MESH] OR compassion fatigue [MESH]) (156,992)

#27 ("0000"[Date - Publication] : "2021/12/01"[Date - Publication]) (33,422,914)

#28 (#3 AND #11 AND #19 AND #26 AND #27) OR (#3 AND #11 AND #19 AND #25 AND #27) (141)

**6. Database: Embase<2011 to 2021>**

[**https://www.embase.com/#advancedSearch**](https://www.embase.com/#advancedSearch)

Search Strategy:

--------------------------------------------------------------------------------

((rural OR countryside OR village) AND (doctor OR physician OR practitioner) AND 'china'/exp AND ('turnover intention'/exp OR 'job dismission tendency'/exp OR 'departure intention tendency'/exp OR 'job demission tendency'/exp) AND 'cross-sectional study'/exp AND [<2011-2021]/py) OR ((rural OR countryside OR village) AND (doctor OR physician OR practitioner) AND 'china'/exp OR 'chinese'/exp AND ('burnout'/exp OR 'burned out'/exp OR ' compassion fatigue '/exp OR 'psychological stress'/exp) AND 'cross-sectional study'/exp AND [<2011-2021]/py) (147)

**7. Web of Science<2011 January 01 to 2021 December 01>**

<https://www.webofscience.com/wos/alldb/summary/3cebb2d7-1c84-46ec-a5e1-f7c0e5637cd9-1afa320b/relevance/1>

Search Strategy:

--------------------------------------------------------------------------------

((TS=(rural OR countryside OR district OR village OR grassroots)) AND (TS=(doctor OR physician OR practitioner OR “health worker” OR “health officer” OR “health personnel” OR “medical personnel” OR “medical worker” OR “medical staff” OR “physician assistant”)) AND (TS=(“turnover intention” OR “departure intention” OR “demission intention” OR “job demission intension” OR “turnover tendency” OR “departure intention tendency” OR “demission tendency” OR “job demission tendency”)) AND (TS=(China OR Chinese))) OR ((TS=(rural OR countryside OR district OR village OR grassroots)) AND (TS=(doctor OR physician OR practitioner OR “health worker” OR “health officer” OR “health personnel” OR “medical personnel” OR “medical worker” OR “medical staff” OR “physician assistant”)) AND (TS=(“burnout” OR “burned out” OR “emotional stress” OR “compassion fatigue” OR “psychological stress”)) AND (TS=(China OR Chinese))) (24)
