# Supplementary figures and images for: Village doctors' dilemma in China: A systematic evaluation of job burnout and turnover intention
Source: Front Public Health. 2022 Nov 10;10:970780. doi: 10.3389/fpubh.2022.970780 (PMC9684668; doi:10.3389/fpubh.2022.970780)

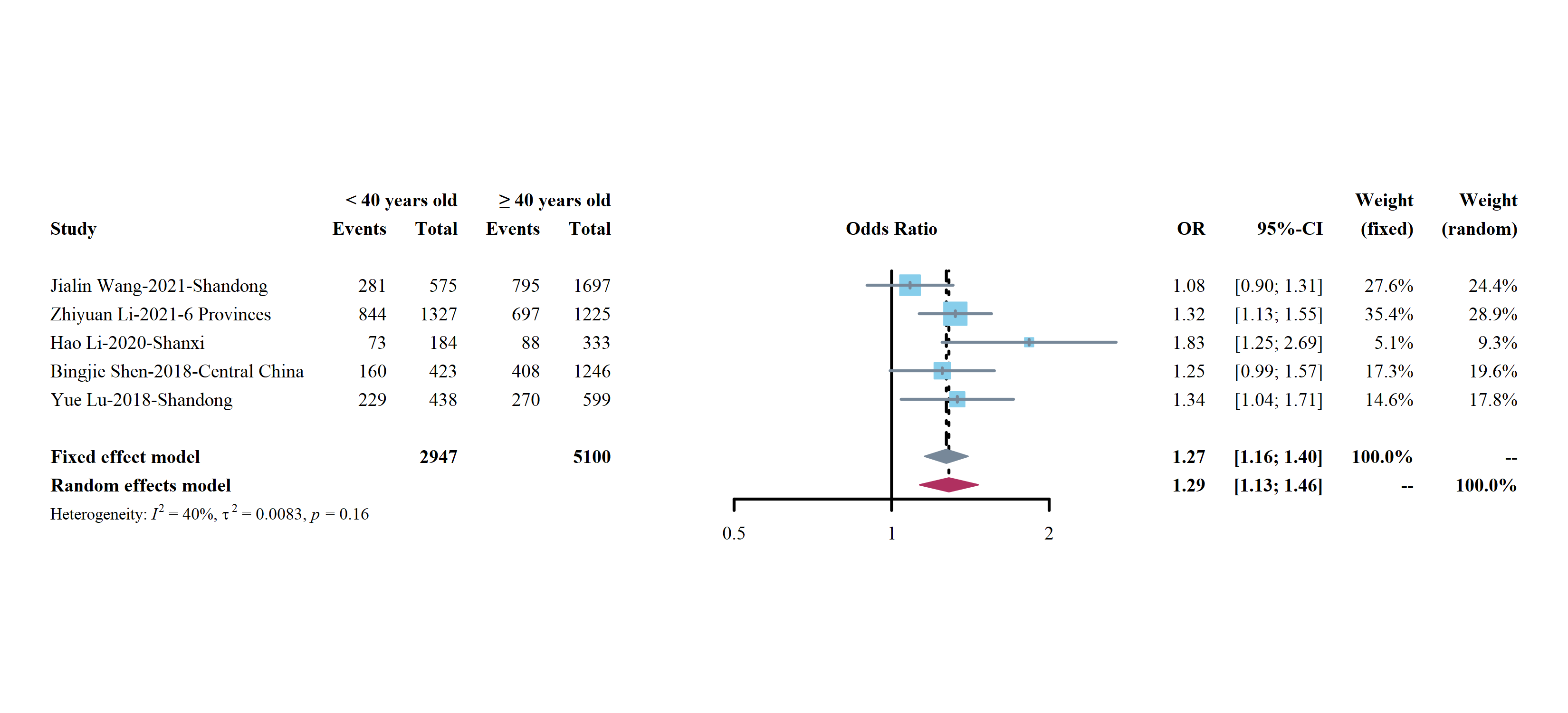

Supplement: Supplementary file 2 [file Data_Sheet_2.ZIP › Appendix C-Forest plots about contributors to turnover intention/Age.png]

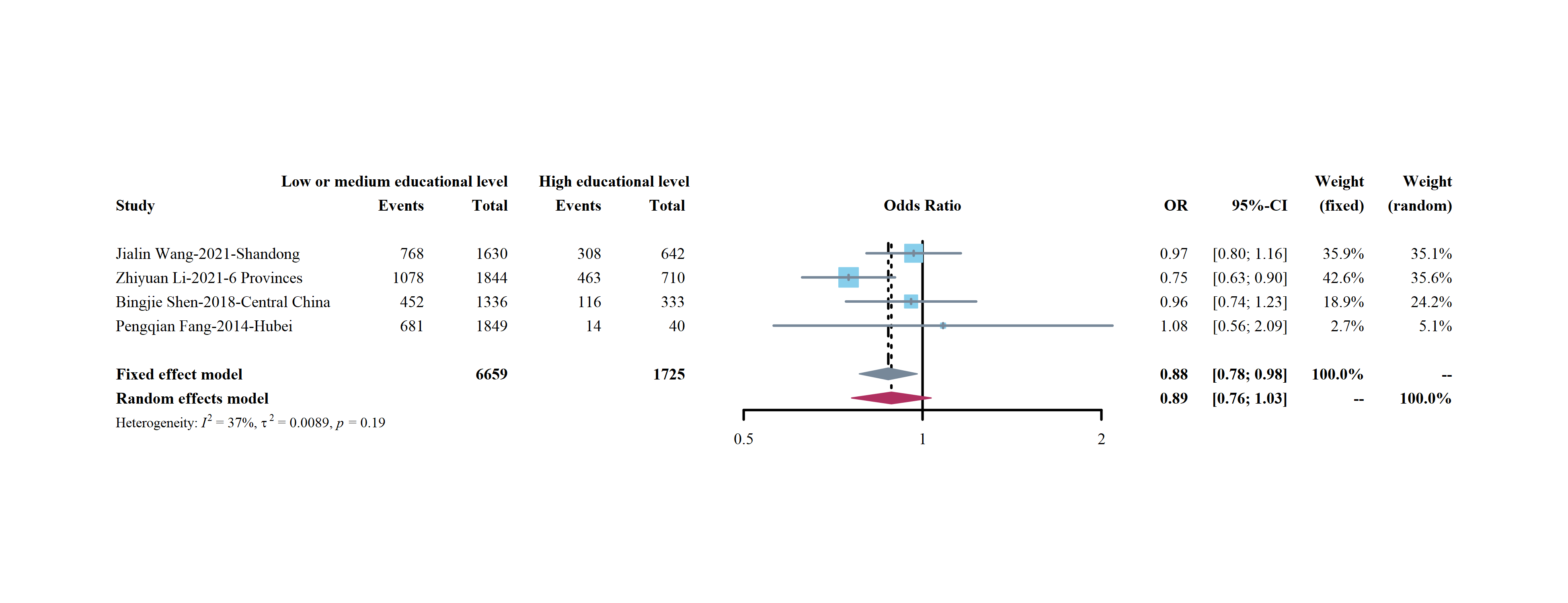

Supplement: Supplementary file 2 [file Data_Sheet_2.ZIP › Appendix C-Forest plots about contributors to turnover intention/Educational level.png]

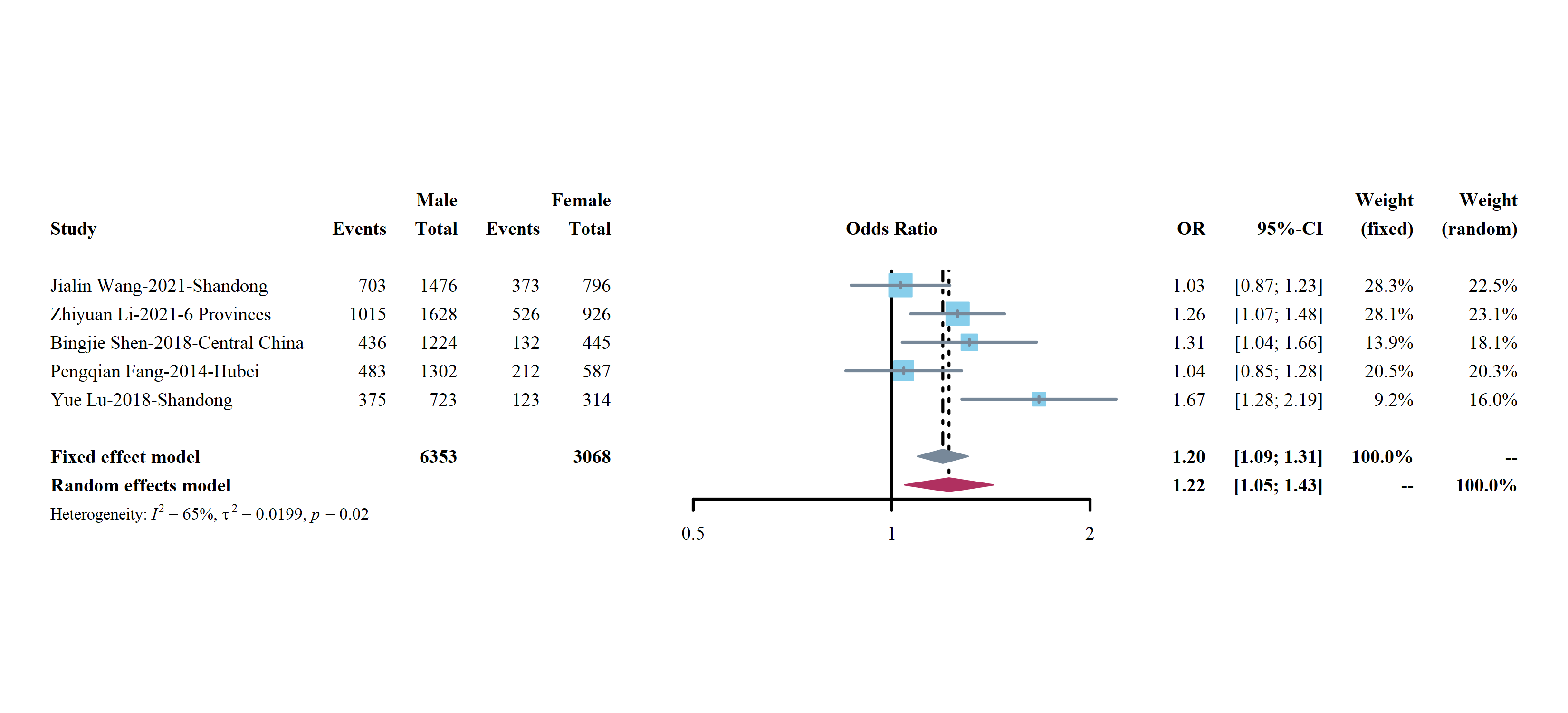

Supplement: Supplementary file 2 [file Data_Sheet_2.ZIP › Appendix C-Forest plots about contributors to turnover intention/Gender.png]

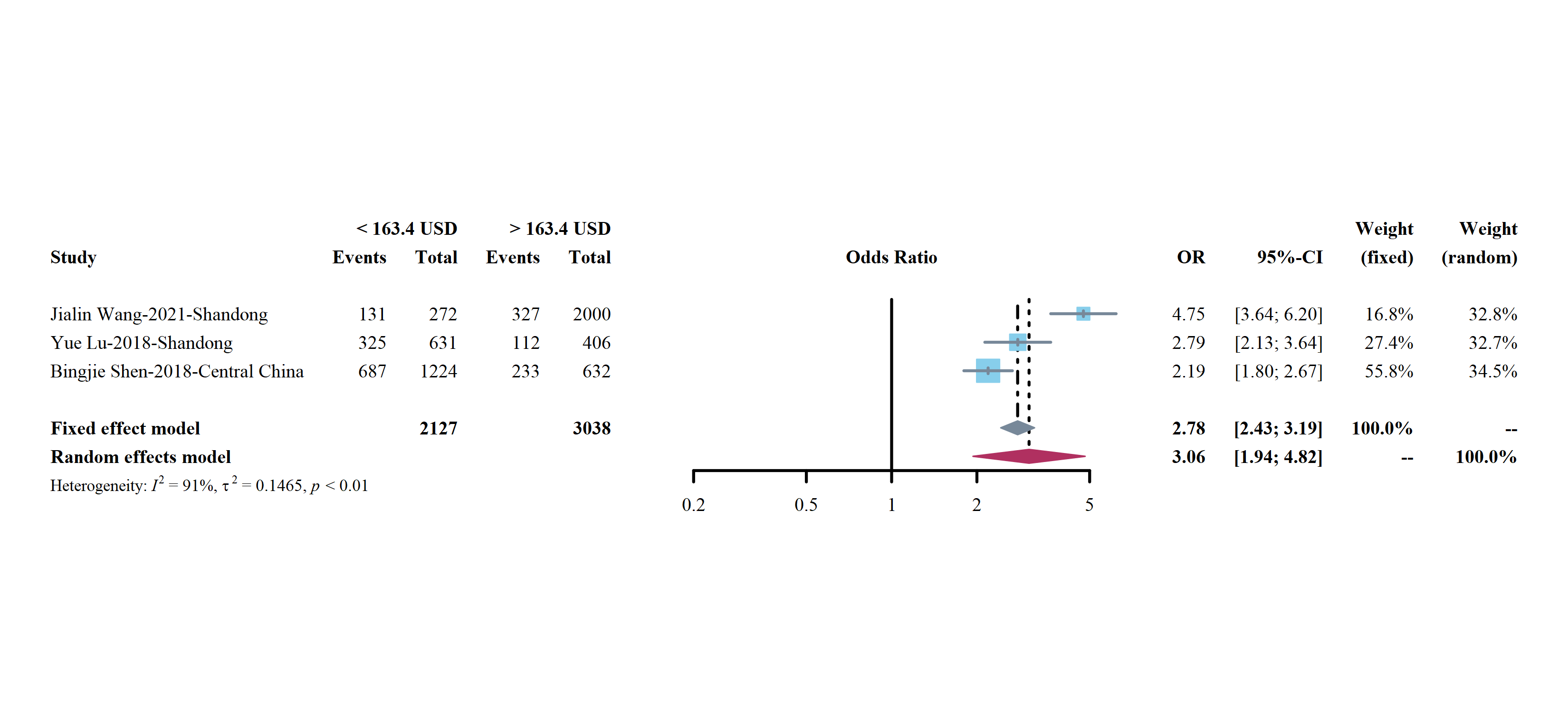

Supplement: Supplementary file 2 [file Data_Sheet_2.ZIP › Appendix C-Forest plots about contributors to turnover intention/Income.png]

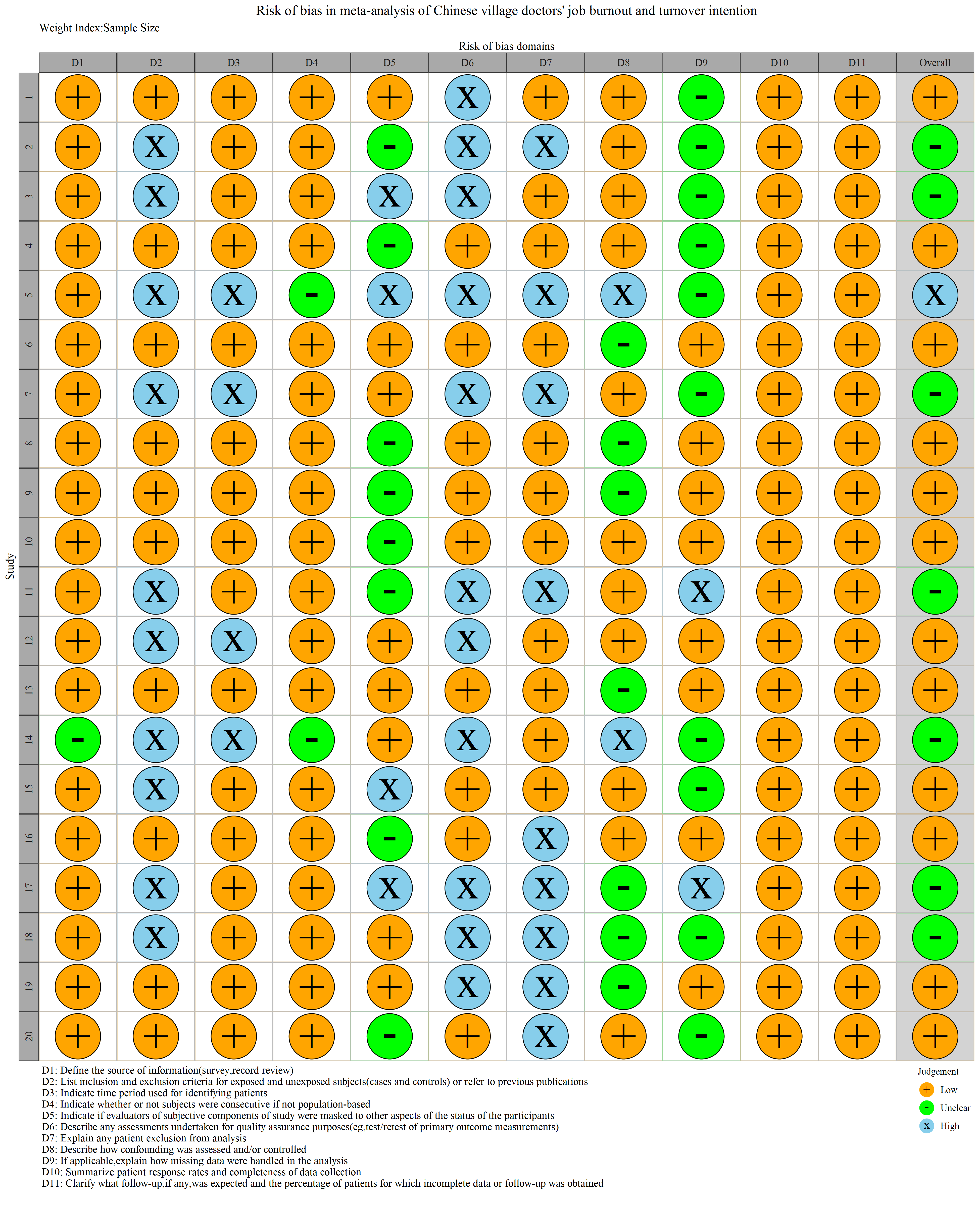

Supplement: Supplementary file 3 [file Image_1.PNG]
